# Graves’ disease coexisting with resistance to thyroid hormone: a rare case

**DOI:** 10.1002/ccr3.1344

**Published:** 2017-12-29

**Authors:** Hongping Sun, Shuhang Xu, Shaofeng Xie, Wen Cao, Guofang Chen, Hongjie Di, Rendong Zheng, Xingjia Li, Xiaodong Mao, Chao Liu

**Affiliations:** ^1^ Endocrine and Diabetes Center Affiliated Hospital of Integrated Traditional Chinese and Western Medicine Jiangsu Province Academy of Traditional Chinese Medicine Nanjing University of Traditional Chinese Medicine Nanjing China

**Keywords:** Genetic mutation, Graves’ disease, thyroid hormone receptor *β*, thyroid hormone resistance

## Abstract

A rare case of resistance to thyroid hormone (RTH) complicated with Graves’ hyperthyroidism was reported. The management of this disease is similar to that of Graves’ disease. Antithyroid drug therapy is the first choice, and iodine therapy and surgery are not recommended due to the possibility of severe hypothyroidism and enlargement of the pituitary gland.

## Introduction

Resistance to thyroid hormone *β* is triggered by a mutated *β*‐isoform of the thyroid hormone receptor. Graves’ disease is a common autoimmune disorder characterized by hyperthyroidism due to circulating autoantibodies. However, resistance to thyroid hormone accompanied by Graves’ disease has been rarely reported. In this report, a 14‐year‐old girl presented with elevated free thyroxine and free triiodothyronine acid concentrations, a decreased serum thyroid‐stimulating hormone concentration, and positivity for thyrotropin receptor antibody. A diagnosis of Graves’ disease was recorded. The patient was treated with oral anti‐hyperthyroidism drugs such as methimazole and propylthiouracil for 3 years, but repeated thyroid function tests during follow‐up continued to show elevated levels of free thyroxine and free triiodothyronine acid along with a normal serum thyroid‐stimulating hormone level. Further gene sequencing tests identified a mutation in the thyroid hormone receptor *β* gene in this patient and her father. Ultimately, the patient was diagnosed with resistance to thyroid hormone complicated by Graves’ disease, which is extremely rare.

Resistance to thyroid hormone (RTH) is an inherited syndrome characterized by reduced target tissue responsiveness to thyroid hormones. It is characterized by elevated serum thyroid hormone (TH) levels and elevated or inappropriately normal thyrotropin levels. Patients with RTH usually exhibit TH resistance in the pituitary and peripheral tissues. The clinical manifestation varies depending on the severity of the mutation and differences in hyposensitivity to TH among individuals as well as in different tissues. While clinical manifestations vary, free thyroxine (FT4) and triiodothyronine three (FT3) levels are always elevated, and the thyroid‐stimulating hormone (TSH) level is normal or mildly elevated and accompanied by thyroid enlargement. Mutation of the thyroid hormone receptor (TR) *β* gene is found in most cases of RTH 1.

The incidence of Graves’ disease in the general population is about 0.5%, accounting for 50%–80% of hyperthyroidism cases. However, the coexistence of Graves’ disease and RTH has been rarely reported. Herein, we report a case of Graves’ disease accompanied by RTH.

## Case Report

Due to recurrent palpitation and tremor over at least 3 years, a 14‐year‐old girl was admitted to our center on 22 July 2013. She had been diagnosed with hyperthyroidism in another hospital according to her thyroid function on 15 July 2010. Methimazole was prescribed primarily, and it was changed for propylthiouracil on 29 June 2011. However, her thyroid function remained abnormal over the 3 years prior to her presentation at our department (Tables 1 and 2). All antithyroid drugs had been suspended for 5 months since 28 January 2013, and she was admitted to our center on 22 July 2013 for further checkup and diagnosis. Her thyroid function was rechecked twice during her stay in our ward. The results showed remarkable elevation of FT3 and FT4 levels with a suppressed TSH level. At both time points, the FT3 concentration exceeded 30.8 pmol/L (normal range, 3.5–6.5 pmol/L), and the FT4 concentration exceeded 154.8 pmol/L (normal range, 11.5–22.7 pmol/L). The serum TSH concentrations at the two time points were 0.013 *μ*IU/mL and 0.006 *μ*IU/mL (normal range, 0.55–4.78 *μ*IU/mL). The measured levels of TSH receptor antibody (TRAb) were 40 mIU/mL and 35.36 mIU/mL (normal range, 0–1.58 mIU/mL). After administration of a small dose of ^131^iodine, the radioactive iodine uptake rate was 68.69% at 2 h (normal range, 4.5%–24.5%), 85.98% at 6 h (normal range, 8.7%–42%), and 69.53% at 24 h (normal range, 19%–61%). A magnetic resonance (MRI) scan taken previously in 2012 at the other hospital showed a slightly enlarged pituitary gland not occupied by a lesion.

**Table 1:** Patient data for measurements taken at Yangzhou Hongquan Hospital (2010.07.15 to 2011.06.04)

Date	FT3 ng/dL	FT4 ng/dL	TSH *μ*IU/mL	Tg ng/mL	Treatment
Normal range	0.202–0.443	0.93–1.71	0.27–4.2	1.4–78	Methimazole, unknown dose
2010‐07‐15	2.78↑	3.17↑	0.037↓	/	Methimazole, unknown dose
2010‐08‐27	1.02↑	2.22↑	0.107↓	5.04	Methimazole, unknown dose
2010‐10‐30	>3.25↑	>7.77↑	0.119↓	10.92	Methimazole, unknown dose
2010‐12‐18	0.508↑	0.78↓	29.96↑	24.29	Methimazole, unknown dose
2011‐01‐20	0.49↑	0.16↓	75.52↑	165.4↑	Methimazole, unknown dose
2011‐02‐11	0.952↑	1.01	20.35↑	138.1↑	Methimazole, unknown dose
2011‐03‐12	0.815↑	0.91↓	13.98↑	183.20↑	Methimazole, unknown dose
2011‐04‐23	0.805↑	1.65	7.05↑	36.52	Methimazole, unknown dose
2011‐06‐04	0.848↑	2.25↑	9.98↑	15.99	Methimazole 5 mg qd

**Table 2:** Patient data for measurements taken at Subei People's Hospital (2011.06.29 to 2012.01.17)

Date	FT3 pmol/L	FT4 pmol/L	TSH *μ*IU/mL	TPOAb U/mL	TgAb U/mL	Treatment
Normal range	3.8–6.0	7.9–14.4	0.34–5.6	0–34	0–115	
2011‐06‐29	9.51↑	27.12↑	2.96	/	/	Propylthiouracil, unknown dose
2011‐08‐01	9.53↑	12.59	9.050↑	/	/	Propylthiouracil, unknown dose
2012‐01‐17	10.26↑	24.6↑	3.83	/	/	Propylthiouracil, unknown dose
2012‐06‐28	11.52↑	19.66	6.37↑	/	/	Propylthiouracil, unknown dose
2012‐08‐03	16.01↑	26.5↑	3.53	/	/	Propylthiouracil, unknown dose
2012‐08‐24	12.72↑	25.05↑	4.04	/	/	Propylthiouracil, unknown dose
2012‐12‐10	16.05↑	31.53↑	2.91	/	/	Propylthiouracil, unknown dose
2013‐01‐28	15.22↑	12.51	5.38↑	>600↑	515.6↑	Treatment was stopped

Physical examination on admission revealed a body temperature of 37.2°C, heart rate of 120 beats per minute, respiratory rate of 18 breaths per minute, blood pressure of 110/60 mmHg (1 mmHg = 0.133 kPa), weight of 40 kg, height 1.55 m, and body mass index of 16.65 kg/m^2^. The patient's thyroid was moderately enlarged without murmur or a nodule. Along with the heart rate of 120 beats per minute, there was obvious accentuation of the first heart sound and tremor, but no edema or proptosis were found. No other specific findings were observed.

The patient's father was advised to undergo a check of his thyroid function and receive genetic testing to determine whether he also had THR, which is a familial disease. Thyroid function tests in our hospital revealed a FT3 concentration of 10.3 pmol/L (normal range, 3.5–6.5 pmol/L), FT4 concentration of 32.03 pmol/L (normal range, 11.5–22.7 pmol/L), and TSH concentration of 1.662 *μ*IU/mL (normal range, 0.55–4.78 *μ*IU/mL) for the father on 05 February 2013 (Table 3). Repeated tests on 22 July 2013 showed elevated FT3 and FT4 levels with a normal serum TSH concentration (Table 3).

**Table 3:** Thyroid function of the patient's father

Date	T3 nmol/l	T4 nmol/l	FT3 pmol/l	FT4 pmol/l	TSH *μ*IU/mL	TgAb U/mL	TPOAb U/mL	Tg ng/mL	TRAb mIU/mL
Normal range	0.92–2.79	24.5–171.6	3.5–6.5	11.5–22.7	0.55–4.78	0–60	0–60	1.4–78	0–1.58
2013‐02‐05	2.33	159.50	10.3↑	32.03↑	1.662	10.9	7.8	94.6↑	/
2013‐07‐22	/	/	9.68↑	33.12↑	2.509	8.1	0.6	/	0.67

**Figure 1:**
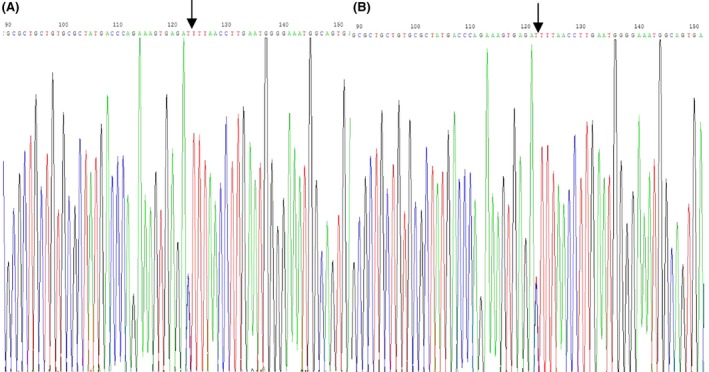
Genetic mutations found in the patient (A) and her father (B). A point mutation in thyroid hormone receptor *β* gene was identified by gene sequencing tests for this patient and her father. The 95th base C was changed to T due to a mutation (C95T) on exon 10 with the codon of ACU changed to AUU, resulting in the encoded amino acid threonine being changed to isoleucine.

The patient's history was not inconsistent with simple Graves’ hyperthyroidism, and her father's thyroid function indicated THR syndrome without any comorbidities. Therefore, genetic testing for mutations of the TR *β* gene was performed. A point mutation in the TR *β* gene was identified in the patient and her father (Fig. 1). The 95th base C was replaced by T (C95T) on exon 10 with the codon of ACU changed to AUU, and the encoded threonine was changed to isoleucine.

Subacute thyroiditis, iatrogenic thyrotoxicosis, iodide‐induced thyrotoxicosis, thyrotropin‐secreting pituitary adenoma, and multinodular toxic goiter were excluded according to the patient's history, clinical manifestations, and follow‐up observations.

The patient was then diagnosed with THR complicated by Graves’ disease. Methimazole 30 mg/day and propranolol 90 mg/day were started on 22 July 2013, and then, methimazole 30 mg/day and propranolol 60 mg/day were maintained from 13 August 2013. After discharge, she was followed up regularly. The patient became asymptomatic with stable thyroid function, slightly elevated FT3 and FT4 levels, and a normal TSH level (Table 4).

**Table 4:** Additional measurements taken during follow‐up (2014.07.22 to 2017.02.06)

Date	FT3 pmol/L	FT4 pmol/L	TSH *μ*IU/mL	TRAb mIU/mL	TPOAb U/mL	TgAb U/mL	Tg ng/mL	Treatment
Normal range	3.5–6.5	11.5–22.7	0.55–4.78	0–1.58	0–60	0–60	1.4–78	
2013‐07‐22	>30.8↑	>154.8↑	0.013↓	>40↑	>1300↑	>500↑	2.5	Methimazole 10 mg tid; Propranolol 30 mg tid
2013‐08‐13	12.82↑	23.13↑	0.017↓	>40↑	>1300	>500↑		Methimazole 10 mg tid; Propranolol 20 mg tid
2013‐10‐07	2.15↓	4.09↓	111.531↑	31.81↑	>1300↑	>500↑	10.1	Methimazole 15 mg qd
2013‐11‐29	10.07↑	19.65	4.593	25.11↑				Methimazole 10 mg qd
2014‐02‐07	13.02↑	37.79↑	0.25↓	19.38↑	>1300↑	>500↑		Methimazole 15 mg qd Propranolol 10 mg tid
2014‐05‐09	10.34↑	25.49↑	3.98	13.82↑				Methimazole 10 mg qd
2014‐07‐04	12.94↑	17.21	2.599	13.19↑	>1300↑			Methimazole 10 mg bid
2014‐08‐11	10.46↑	16.52	12.711↑	9.74↑	>1300↑	>500↑		Methimazole 15 mg qd
2015‐02‐09	9.85↑	24.63↑	4.148	3.99↑				Methimazole 10 mg qd
2015‐08‐10	10.15↑	36.53↑	2.989	4.26↑	>1300↑	>500↑		Methimazole 10 mg tid Thalidomide 50 mg bid
2015‐10‐06	13.51↑	15.01	5.435↑	6.47↑	>1300↑	>500↑		Methimazole 10 mg bid Thalidomide 50 mg bid
2016‐01‐26	7.05↑	5.27↓	69.768↑	2.45↑				Methimazole 15 mg qd Thalidomide 75 mg qn
2016‐05‐30	10.83↑	13.92	8.621↑	1.74↑				Methimazole 15 mg qd Thalidomide 50 mg qn Methylprednisolone 4 mg qd
2016‐08‐15	11.12↑	25.72↑	5.433↑	0.64				Methimazole 15 mg qd
2016‐11‐21	11.71↑	29.85↑	2.762	<0.3				Methimazole 15 mg qd
2017‐02‐06	11.59↑	38.43↑	3.007	1.69↑	>1300↑	>500↑		Methimazole 10 mg bid

## Discussion

Resistance to thyroid hormone (RTH) complicated by Graves’ disease has been rarely reported. The optimal therapy for this complex situation remains unknown. Treatment with anti‐hyperthyroidism drug remains the primary choice for Graves’ hyperthyroidism complicated by RTH. Radioactive iodine therapy and thyroidectomy are not commonly recommended for pituitary enlargement caused by TH deficiency. This disease can be well controlled by anti‐hyperthyroidism drugs, but no unified criteria exist based on clinical signs and symptoms and thyroid function. The optimal treatment should only slightly increase FT3 and FT4 levels, normalize the TSH level, and avoid drug overdose.

One case of hyperthyroidism complicated by THR was reported by Sato et al. in 2010 2. The symptom of hyperthyroidism was relieved after treatment with methimazole for 10 months. Their patient then tested negatively for anti‐TSH receptor antibody (TRAb) and thyroid‐stimulating antibody (TSAB) in the following 2 years with normal FT3 and TSH levels and a slightly increased FT4 level. The mutated gene was P453T. Sivakumar 3 reported a similar case in which hypothyroidism appeared after treatment with ^131^I and 325 *μ*g levothyroxine. Ogawa et al. 4 reported one case of RTH combined with Graves’ hyperthyroidism in a patient with the A234T mutation in the TR *β* gene. A mutation at codon 251 causing replacement of a glycine (G) with arginine (R) was reported by Shiwa et al. 5 in another case. Most patients with RTH have demonstrated a TR *β* gene mutation, but for some patients, no mutations could be detected and the molecular mechanism of their condition is still unclear.

In this case, the TR *β* gene of the patient and her father were mutated (C95T), which indicated a familial condition. During follow‐up, the patient's status remained relatively stable, and the dose of methimazole was adjusted according to the follow‐up observations and reported symptoms. The detailed follow‐up results are provided in the Additional Information.

## Conclusion

This paper reports a rare case of resistance to thyroid hormone complicated by Graves’ hyperthyroidism. During the follow‐up, we found the patient's status was stable with drug therapy. Thus, iodine therapy and surgery are not recommended, because they could induce severe hypothyroidism and hyperplasia of the pituitary gland.

## Authorship

HS: in charge of follow‐up. SX, WC, and GC: helped modify the case report. SX, HD, and RZ: helped make the diagnosis and the treatment regimen. XL and XM: experimented the gene test.

## Conflict of Interest

None declared.
